# Explaining urban street perception inequities between residents and tourists using interpretable machine learning

**DOI:** 10.1371/journal.pone.0345073

**Published:** 2026-03-17

**Authors:** Baoyue Kuang, Hao Yang, Yu Zhu, Zeyuan Chang

**Affiliations:** 1 Department of Landscape Architecture, Kyungpook National University, Daegu, South Korea; 2 Department of Interior Environmental Design, Pusan National University, Busan, South Korea; 3 School of Architecture, Southeast University, Nanjing, China; East China Normal University, CHINA

## Abstract

Understanding how different social groups perceive urban streets is essential for inclusive and sustainable urban design. This study proposes an interpretable and scalable machine learning framework that integrates Street View Images with subjective evaluations to examine perceptual differences between residents and tourists. Using data from Xi’an’s historic Mingcheng District, we collected perception ratings across five dimensions-safety, comfort, convenience, pleasure, and sociability-and analyzed how visual and environmental features shape these perceptions. The framework combines predictive modeling and explainable analysis to uncover both linear and nonlinear drivers of perception. The results show that tourists are more responsive to symbolic and aesthetic cues, while residents emphasize functional and comfort-related features. Key visual elements such as vegetation, building facades, and spatial openness exert different effects on the two groups. By revealing these perceptual disparities, the study provides actionable insights for perception-informed and equitable street design strategies that better address the needs of diverse urban users.

## 1. Introduction

Public spaces play a crucial role in shaping vibrant urban experiences [1]. Urban streets are not only channels for transportation but also vital public spaces that support both daily life and tourism activities [2]. The perceptual quality of street environments directly influences travel behavior [3], emotional well-being [4], and social interaction [5], serving as a key indicator of urban livability, inclusiveness, and equity. [6] In recent years, with the rise of human-centered design principles, researchers have increasingly emphasized user perspectives in evaluating street environments [7]. In tourism-oriented cities, streets often serve a dual function, facilitating local commuting while also shaping tourists’ first impressions [8]. However, differences in spatial roles, behavioral patterns, and psychological expectations between residents and tourists frequently lead to divergent perceptions of the same street environment [9]. Neglecting such differences can result in a homogenized spatial design that overlooks the experiential needs of specific user groups. Therefore, identifying perceptual differences between residents and tourists from the perspective of spatial equity and perceptual diversity has become an emerging topic in public space research [5,10,11].

Urban perception is inherently interdisciplinary, drawing upon theories from environmental psychology, architecture, landscape studies, and urban design. Classic psychological frameworks such as Gibson’s environmental affordance theory [12] and Kaplan and Kaplan’s preference model [13] emphasize how individuals extract functional cues, visual coherence, and restorative qualities from physical environments. Architectural and spatial cognition research also highlights how enclosure, façade articulation, and spatial legibility shape experiential responses to streetscapes [14,15]. Meanwhile, urban design theories—from Lynch’s concept of imageability [16] to Gehl’s human-scale principles [17]—demonstrate how built form, social interaction, and spatial configuration influence comfort, sociability, and behavioral patterns. Landscape studies further show that vegetation and natural elements enhance psychological well-being and visual preference in urban spaces [18]. More recently, computational and AI-driven approaches have integrated these perspectives by linking visual semantics extracted from images with human perceptual judgments [19,20]. Together, these interdisciplinary foundations suggest that street perception is not merely a visual outcome but an interaction between physical form, cognitive processing, and social roles, thereby underscoring the need for a multi-domain analytical framework. However, despite these theoretical advances, empirical studies have struggled to operationalize these concepts at scale, particularly when comparing different user groups. This challenge has driven a methodological shift from traditional perception measurements toward image-based and data-driven approaches capable of capturing multi-group perceptual variation across urban contexts. Classic techniques like subjective questionnaires [15], activity diaries [21], and walk audits [22] offer in-depth insights but are difficult to scale for large, multi-group urban contexts. With the growing availability of Street View Images (SVI), convolutional neural networks, and artificial intelligence algorithms, image-based perception modeling has become mainstream [23,24], enabling high resolution and fine-grained urban perception analysis at relatively low cost. However, existing studies have mostly focused on single user groups such as residents, older adults, or children, while largely overlooking tourists as key urban users [7,25,26]. Furthermore, current perception modeling often lacks attention to user identity and semantic differences, limiting its applicability to inclusive design practices.

To enable scalable and interpretable perception modeling, recent studies have adopted machine learning methods such as random forests, gradient boosting trees, and deep neural networks to predict the relationship between visual features and perceptual ratings extracted from SVI. [27,28]. While these models improve predictive performance, they often operate as ‘black boxes’, making it difficult to explain how decisions are made or why certain features matter. [29] To address this challenge, explainable AI techniques such as SHapley Additive Explanations (SHAP) have been introduced to reveal both global and local marginal effects of variables on perception outcomes [30,31]. SHAP has been widely applied to interpret model outputs related to street safety perception [32], happiness [33], and multi-dimensional urban perception [34], significantly improving model transparency and practical relevance. However, these approaches remain largely limited to single user groups, lacking transferable modeling frameworks that accommodate multiple types of users. In summary, although existing street perception research has made theoretical and methodological progress, three major limitations remain: First, most studies have focused on a single user group, with minimal inclusion of or comparison with tourists, thereby limiting their applicability in tourism-driven cities and mixed-use urban zones [5,35]. Second, there is a disconnection between prediction and explanation—explanatory analyses often rely on simple feature ranking and lack insights into causal mechanisms or group-specific explanatory pathways [7,36]. Third, few studies explore spatial structures or spatial heterogeneity between groups. While some have employed spatial autocorrelation and hotspot analysis, these efforts often remain at the level of descriptive mapping [37,38]. These limitations reveal that existing approaches tend to treat perception as a homogeneous cognitive response, overlooking how individual roles, contextual experience, and spatial configurations jointly shape urban perception. To advance the theoretical understanding of urban perception, this study introduces a role-based cognitive perspective that explicitly distinguishes between residents and tourists as distinct perceptual agents. This conceptual lens highlights that perception is not only visually driven but also socially and functionally conditioned by users’ spatial engagement and purpose of stay. Methodologically, by integrating semantic segmentation of street view imagery, interpretable machine learning (Random Forest, ElasticNet, XGBoost, and SHAP), and spatial autocorrelation analysis, the proposed framework bridges the gap between prediction and explanation. It enables a multi-level examination of perception – from visual semantics to spatial clustering – thereby enhancing the explanatory depth and spatial interpretability of perceptual modeling. This integrative approach allows for a more comprehensive exploration of how urban form, visual composition, and user identity interact to produce differentiated perceptual outcomes, overcoming prior studies’ constraints of single-group focus, weak causal interpretability, and limited spatial contextualization.

**Fig 1:**
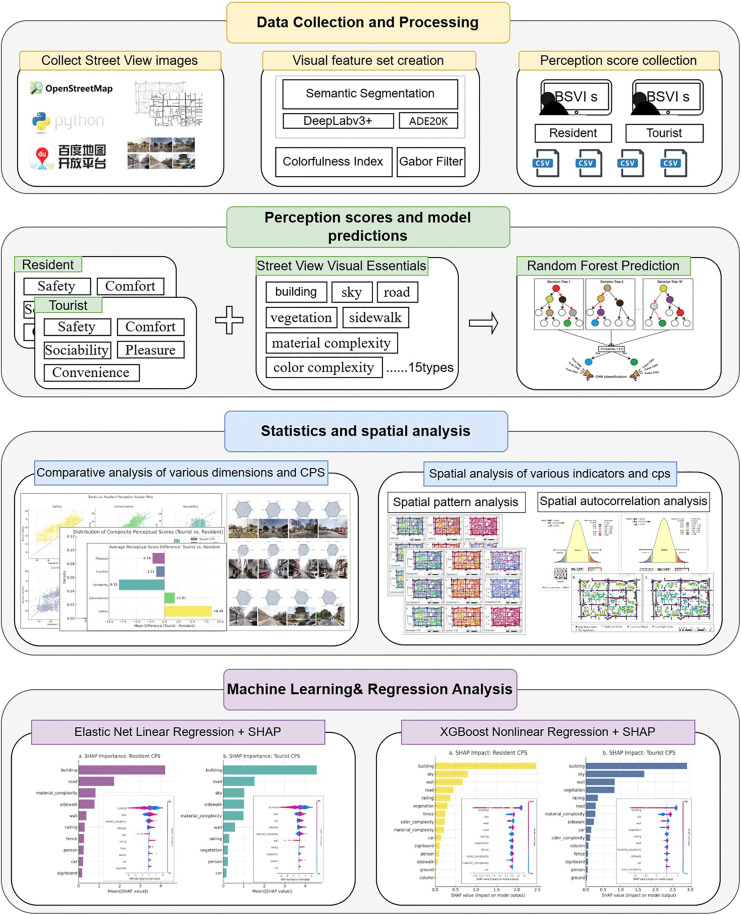
Research framework and technical route.

In response, this study proposes an interpretable perception modeling framework that combines SVI, machine learning, and spatial analysis to identify perceptual differences between residents and tourists in urban street environments and to explore their underlying mechanisms. The historic core of Xi’an, China, was selected as the empirical study area due to its dual spatial characteristics, it is a high-density urban core with overlapping networks of everyday life and intensive tourist activities. A total of 5,309 SVIs were collected, and 30 volunteers (15 residents and 15 tourists) were recruited to provide multidimensional subjective evaluations. The study design consists of four stages: (1) data collection and visual feature extraction via semantic segmentation and image complexity analysis; (2) training Random Forest models to predict residents’ and tourists’ composite perceptual scores; (3) conducting statistical and spatial clustering analyses (moran’s I and LISA) to identify spatial heterogeneity; and (4) applying Elastic Net Regression(ENR) and extreme gradient boosting (XGBoost) regression models with SHAP analysis to interpret the influence pathways and relative weights of different visual features across user groups. The overall workflow is illustrated in Fig 1.

## 2. Methods

### 2.1. Study area

**Fig 2:**
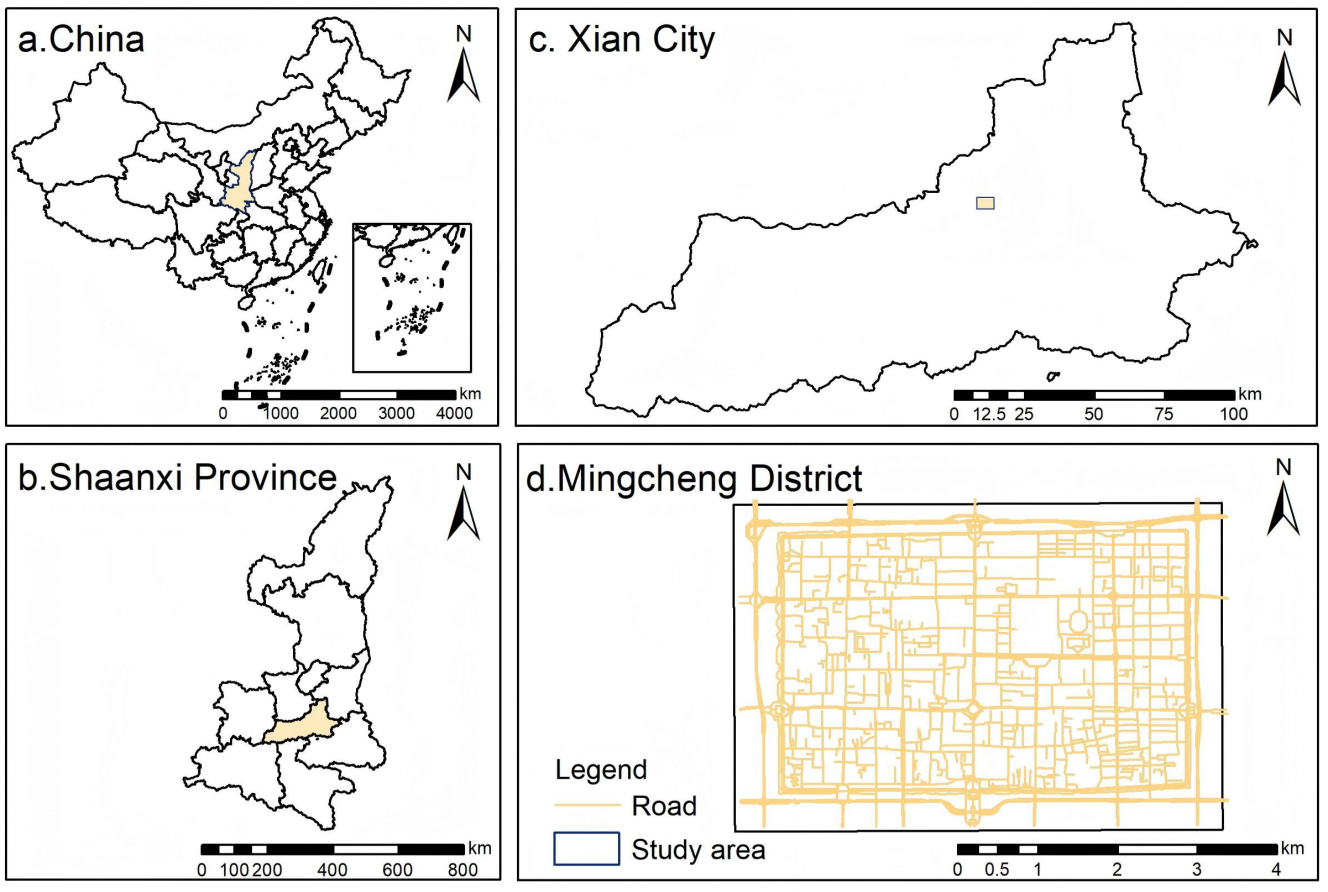
Location and road network of the study area.

This study selects the Mingcheng District, the historic core area of Xi’an, China, as the research site (Fig 2). Surrounded by the ancient city walls, this central urban area exemplifies a ‘dual-function’ urban zone, characterized by both high-density residential life and intense tourism activities. According to the 2022 statistical report, the district has a resident population of approximately 306,462 and covers an area of around 1,710 hectares (Source: Xi’an bureau of statistics, http://tjj.xa.gov.cn/tjnj/2023/zk/indexch.htm; accessed December 3, 2024). During the 2025 may day holiday, the Xi’an city wall scenic area received over 188,000 visitors in a single day, while other key tourist destinations such as the bell and drum tower museum and the Xi’an incident memorial attracted more than 100,000 visitors each (data source: cultural relics shaanxi, https://mp.weixin.qq.com/s/2Ye9EFCh46WfkBdc-f7t9A; accessed may 6, 2025). Within the district, residential alleys, cultural streets, historical sites, and commercial arteries are intricately interwoven, forming a complex urban spatial structure. This high overlap of everyday life and temporary tourist behavior makes the area an ideal setting for investigating perceptual differences between residents and tourists in urban street environments.

### 2.2. Data

#### 2.2.1. Street view image collection.

To comprehensively represent the visual characteristics of urban streets, we generated equidistant spatial sampling points at 50-meter intervals across the study area using open road network data from OpenStreetMap (OSM, https://www.openstreetmap.org). SVIs were collected via the Baidu Maps API (https://lbsyun.baidu.com/), with each location captured at a resolution of 3600 × 600 pixels under a 360-degree horizontal field of view. Four directional views (0°, 90°, 180°, and 270°) were stitched together to ensure spatial integrity and completeness. Following the acquisition, all SVIs were manually reviewed by two researchers to eliminate images taken in underground tunnels, private alleys, or those with severe overexposure, blur, or occlusion. The final dataset consists of 5,309 valid SVIs. This dataset covers approximately 89.7% of the street network within the study area, providing a solid foundation for subsequent modeling and analysis [39].

#### 2.2.2. Visual feature extraction.

**Fig 3:**
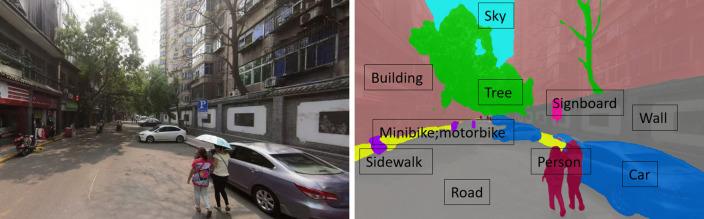
Illustration of semantic segmentation results.

To extract structured and objective visual information from SVIs, we employed the DeepLabv3 + semantic segmentation model, which is based on a deep convolutional neural network architecture. DeepLabv3 + addresses the common trade-off between accuracy and efficiency found in earlier approaches [40]. Trained on the ADE20K dataset, a widely used benchmark for urban scene understanding, the model demonstrated strong multi-class semantic recognition capability, accurately classifying pixels into typical urban visual categories such as buildings, sky, roads, sidewalks, vegetation, vehicles, and walls [41]. An example of the semantic segmentation output is provided in Fig 3 to illustrate how categories such as building and wall are distinguished in practice. In our experiments, the model achieved an average pixel accuracy of 91.83% on the training set and 89.23% on the validation set. These outputs were converted into semantic feature vectors by calculating the pixel proportion of each visual element. All features were normalized before being input into the Random Forest models for perceptual score prediction [39].

In addition, to characterize the visual complexity of the images, we calculated both color complexity and material complexity. Urban color and material composition significantly affect not only the aesthetic expression of space but also the psychological perception of both residents and tourists [42,43]. Color complexity was measured by counting the number of unique RGB color combinations after image simplification, reflecting the diversity of colors present in the scene [44]. Material complexity was computed from grayscale versions of the images using the Sobel operator to extract edge information; texture edge gradients were then used to quantify the structural intricacy of surface textures [45]. Ultimately, we constructed a feature vector system composed of 15 semantic and visual statistical features, which served as input variables for predicting perceptual evaluations.

#### 2.2.3. Perception data collection.

The perceptual evaluation framework adopted in this study draws from established theoretical systems in urban design and public space research. Participants were asked to rate each SVI based on five perceptual dimensions: safety, comfort, convenience, pleasure, and sociability. These dimensions were designed to comprehensively capture both the functional characteristics and experiential quality of urban streets. To support holistic modeling and comparative analysis, these five dimensions were later integrated into a single composite perceptual scores, calculated as a weighted combination of the individual scores. The weighting and validation of composite perceptual scores are detailed in Section 4.2.1. In developing the perceptual framework, we drew from theories developed by Jan Gehl and the Project for Public Spaces (PPS). In cities for people (2010), Gehl emphasizes that the quality of public space depends on meeting a hierarchy of human needs [17]: necessary activities (e.g., commuting, errands) require spaces to ensure safety and accessibility; optional activities (e.g., walking, lingering) rely on comfort and aesthetic appeal; and social activities (e.g., communication, observation, performance) build upon the previous two, depending further on a sense of place and opportunities for interpersonal connection [46,47]. Within this hierarchical model, sociability represents the highest level of perception, indicating that streets should not only be safe and walkable, but also vibrant and socially engaging.

Similarly, PPS’s ‘great public spaces’ model identifies sociability as one of its four core dimensions, alongside accessibility, activity, and comfort. According to PPS, the key to evaluating a space lies in how much social interaction it fosters [48]. While both frameworks have been widely adopted in urban design and public space research, they were originally conceptualized from a resident-centered perspective, focusing on long-term users’ needs and behaviors. In tourism-oriented contexts, these models may not fully capture the short-term, visually-driven, or symbolic preferences of tourists. Nevertheless, their structured hierarchies of spatial use and emphasis on social interaction provide a flexible foundation for adapting perceptual dimensions to diverse user groups. This study acknowledges these limitations and adapts the original dimensions with careful operationalization to ensure applicability across residents and tourists. For example, ‘pleasure’ was included as a distinct dimension to reflect the emotional and aesthetic responses often prioritized by tourists, while ‘convenience’ was refined to address accessibility from both local and outsider perspectives. Through this adaptation, the five perceptual dimensions were operationalized in this study as follows: Safety, The perceived sense of security, including freedom from threats such as traffic conflicts, poor lighting, or unsafe design; Comfort, The perceived ease of the street space, shaped by intuitive impressions such as cleanliness, greenery, spatial openness,and walkability; Convenience, The ease of accessing nearby transport, services, or street connectivity; Pleasure, Visual appeal and emotional enjoyment, reflecting environmental attractiveness and aesthetic qualities; Sociability, The space’s capacity to promote interpersonal interaction, foster community belonging, and exhibit a strong sense of place. To construct a comparative dataset of resident and tourist perceptions, we recruited 30 volunteers—15 local residents and 15 short-term tourists. A purposive sampling method was used to ensure all participants had prior spatial experience with the study area. Residents were required to reside within the district, while tourists must have visited within the past 12 months to ensure their perceptual impressions were recent and valid. All participants were unaffiliated with the research team and provided informed consent. Informed consent was obtained electronically through the online survey interface, where participants confirmed their voluntary participation by clicking the ‘Start’ button after reading the study instructions.

The perception rating process was conducted in two rounds, during July 2024 and April 2025, respectively. A stratified sampling algorithm was applied to randomly select 500 panoramic SVIs from across the study area. Each participant was assigned 50 images, with each image rated by at least two individuals from different user groups to improve cross-validation and ensure balanced group representation for subsequent modeling. Ratings were completed through an online platform, with participants instructed to rate each image based on their first impressions across the five dimensions. Prior to scoring, standardized definitions and sample images were presented by the research team to calibrate participant understanding. All images were previewable to ensure participants were familiar with the diversity of street environments. Following the methodology of Yao and Han [27,49], participants used a 0–100 scale, where 0 indicated complete disagreement and 100 indicated high agreement with the perceptual trait. To ensure intuitive impressions, each image had to be viewed for no less than 10 seconds before scoring [50]. All ratings were compiled into an annotated dataset for training the random forest prediction models. To assess internal consistency, McDonald’s omega (ω) and Cronbach’s alpha were calculated for each dimension, with both exceeding 0.8, indicating strong reliability and internal agreement in the perception ratings.

### 2.3. Urban perceptual score prediction using random forests

To predict street-level perceptual scores, we adopted a machine learning method based on Random Forest model. Random forests are ensemble learning algorithms composed of multiple decision trees and are known for their high accuracy and robustness when modeling nonlinear relationships between high-dimensional features and perceptual responses [27,51]. This method has been widely applied in prior perceptual modeling research and is particularly suitable for capturing complex patterns within semantically segmented SVI data.

During model training, each decision tree is constructed using two-thirds of the samples randomly selected with replacement, while the remaining one-third serves as out-of-bag (OOB) samples. These OOB samples are used to estimate generalization error and compute feature importance. The importance of a variable ${\text{X}}_{\text{j}}$ in tree n is calculated based on the decrease in model accuracy when the values of ${\text{X}}_{\text{j}}$ are randomly permuted among OOB samples. The formal definition is as follows:


$${\text{V}}_{\text{n}}({\text{X}}_{\text{j}})=\frac{\text{1}}{{\text{N}}_{\text{OOB}}}\left(\sum_{\text{i}=\text{1}}^{{\text{N}}_{\text{OOB}}}\text{I}\left[\text{f}({\text{X}}_{\text{i}})={\text{f}}_{\text{n}}({\text{X}}_{\text{i}})\right]-\sum_{\text{i}=\text{1}}^{{\text{N}}_{\text{OOB}}}\text{I}\left[\text{f}({\text{X}}_{\text{i}})={\text{f}}_{\text{n}}({\text{X}}_{\text{i}}^{\prime })\right]\right)$$ (1)

Where,${\text{X}}_{\text{j}}$: the j th input feature used in model training; ${\text{X}}_{\text{i}}$: the original OOB sample; ${\text{X}}_{\text{i}}^{\prime }$: the OOB sample after random permutation of feature${\text{X}}_{\text{j}}$; $\text{f}\left({\text{X}}_{\text{i}}\right)$: the true label (i.e., perceptual score); ${\text{f}}_{\text{n}}\left({\text{X}}_{\text{i}}\right)$: the prediction result of the nth tree; I[·]: indicator function that returns 1 if the prediction is correct and 0 otherwise; ${\text{N}}_{\text{OOB}}$: the number of OOB samples in the nth tree.The variable importance score$\text{V}{\text{I}}_{\text{n}}\left({\text{X}}_{\text{j}}\right)$eflects the extent to which randomizing feature ${\text{X}}_{\text{j}}$ reduces model prediction accuracy. The final importance of a variable is derived by averaging its importance scores across all trees.The final model was trained using an 80:20 split of the dataset into training and testing sets, with five-fold cross-validation used to tune hyperparameters. Model performance was evaluated using mean absolute error (MAE), root mean square error (RMSE), and coefficient of determination (R²), which are widely adopted for perceptual prediction tasks due to their interpretability and robustness [52].

### 2.4. Spatial autocorrelation analysis

To examine whether the composite perceptual scores exhibits spatial clustering or heterogeneity, we conducted a spatial autocorrelation analysis. This method evaluates the degree of correlation between composite perceptual scores values of nearby street segments, allowing for a deeper understanding of the potential spatial structure of the urban environment [37,38]. We first calculated the global Moran’s I to assess the overall spatial autocorrelation within the study area. The formula is as follows:


$$I=\frac{n}{W}\cdot \frac{\sum_{i=\text{1}}^{n}\sum_{j=\text{1}}^{n}w_{ij}(x_{i}-\bar{x})(x_{j}-\bar{x})}{\sum_{i=\text{1}}^{n}(x_{i}-\bar{x}{)}^{\text{2}}}$$ (2)

Where, ${\text{x}}_{\text{i}}$，${\text{x}}_{\text{j}}$: predicted composite perceptual scores at locationsi and j; $\bar{\text{x}}$: mean composite perceptual scores across all nsamples; ${\text{w}}_{\text{ij}}$: spatial weight between locations i and j; $\text{W}=\sum \text{i}\cdot \sum \text{j }\cdot {\text{w}}_{\text{ij}}$:sum of all spatial weights.

Moran’s I ranges from −1 to +1. Positive values indicate clustering of similar values, negative values indicate dispersion, and values near zero suggest spatial randomness. In this study, we used a row-standardized spatial weight matrix based on street segment adjacency and a fixed bandwidth distance to define the neighborhood. This method reflects how proximity and connectivity within the urban street network influence perceptual similarities, offering a bottom-up spatial perspective.

To further explore localized spatial anomalies, we applied Local Indicators of Spatial Association (LISA) to identify spatial clusters and local outliers. Each street segment was categorized into one of four types: high-high (HH), low-low (LL), high-low (HL), and low-high (LH), based on its composite perceptual scores value and the average composite perceptual scores values of its neighbors. These four categories represent clustered high scores, clustered low scores, and spatial outliers. We visualized LISA cluster maps for both resident and tourist composite perceptual scores predictions to compare spatial perceptual differences across groups. By identifying areas of convergence, mismatch, or conflict, this analysis provides spatial insights for perception-sensitive and group-specific public space interventions.

Such spatial analysis reveals the spatial patterns behind perceptual differences, complementing predictive modeling and bridging the gap between image-based modeling and neighborhood-scale urban design.

### 2.5. Modeling perceptual influence mechanisms

#### 2.5.1. Elastic net regression.

To interpret the contribution of visual features to the predicted composite perceptual scores, we employed ENR as a linear baseline model. ENR is a regularization technique that combines the advantages of both Lasso and Ridge regression by introducing L1 and L2 penalties into the objective function. This hybrid regularization allows the model to handle multicollinearity and perform feature selection simultaneously, making it highly suitable for high-dimensional prediction tasks based on image data [53]. The training dataset is denoted as $\text{D}=\{({\text{x}}_{\text{i}},{\text{y}}_{\text{i}}{\}}_{\text{i}=\text{1}}^{\text{N}}$，where ${\text{x}}_{\text{i}}$represents the 16-dimensional visual feature vector of the ith SVI, and ${\text{y}}_{\text{i}}$ enotes the corresponding composite perceptual scores value. The objective of ENR is to minimize the regularized least squares loss function:


$$\underset{\beta }{\text{min}}\left\{\frac{\text{1}}{\text{2N}}\sum_{\text{i}=\text{1}}^{\text{N}}({\text{y}}_{\text{i}}-{\text{x}}_{\text{i}}^{⊤}\beta {)}^{\text{2}}+\lambda \left[\alpha ||\beta |{|}_{\text{1}}+\frac{\text{1}-\alpha }{\text{2}}||\beta |{|}_{\text{2}}^{\text{2}}\right]\right\}$$ (3)

Where, β denotes the vector of regression coefficients; λ controls the overall strength of the regularization; α∈[0,1] adjusts the balance between the L1 and L2 penalties. When α=1 , the model becomes equivalent to Lasso regression; when α=0 , it corresponds to Ridge regression. Elastic Net flexibly integrates both penalties depending on the data, enabling efficient feature selection and shrinkage.

#### 2.5.2. Extreme gradient boosting regression.

To capture potential nonlinear relationships and feature interactions that linear models may fail to identify, we employed XGBoost as a nonlinear modeling approach. XGBoost is an ensemble learning algorithm based on gradient-boosted decision trees, which iteratively constructs models to minimize a differentiable loss function. Due to its scalability, regularization capacity, and ability to model feature interactions, XGBoost has demonstrated strong performance in structured data prediction tasks [54].

After t rounds of boosting, the prediction for sample i is formalized as:


$${\hat{y}}_{i}^{\left(t\right)}=\sum_{k=\text{1}}^{t}f_{k}({𝐱}_{i}),\;f_{k}\in F$$ (4)

Where, ${\hat{\text{y}}}_{\text{i}}^{\left(\text{t}\right)}$: prediction at iteration t; ${\text{f}}_{\text{k}}$: the prediction function of the kth regression tree; F: the space of regression trees.The training objective includes a regularized loss function defined as:


$$L^{\left(t\right)}=\sum_{i=\text{1}}^{N}l(y_{i},{\hat{y}}_{i}^{\left(t\right)})+\sum_{k=\text{1}}^{t}\Omega (f_{k}),\;\Omega (f)=\gamma T+\frac{\text{1}}{\text{2}}\lambda ||w|{|}_{\text{2}}^{\text{2}}$$ (5)

Where,l(·): the loss function; $\Omega ({\text{f}}_{\text{k}})$: the regularization term;T: the number of leaves in the tree; $||\text{w}|{|}_{\text{2}}^{\text{2}}$: the L2 norm of the leaf weights; γ and λ: regularization parameters that control model complexity. XGBoost models were trained separately for residents and tourists to predict their composite perceptual scores values. Its ability to learn nonlinear feature contributions and interactions complements the ENR model and provides a foundation for interpreting model outputs through SHAP analysis.

#### 2.5.3. Model interpretation using SHapley Additive exPlanations.

To enhance the transparency of the predictive models and understand the marginal effect of each visual feature on the predicted perceptual score, we applied SHapley Additive exPlanations (SHAP). SHAP is a game-theoretic framework that attributes the output of a machine learning model to its input features by quantifying the contribution of each feature to a specific prediction [55]. The SHAP value decomposition is defined as:


$$f(𝐱)=\varphi_{\text{0}}+\sum_{j=\text{1}}^{p}\varphi_{j}$$ (6)

Where, $\varphi_{\text{0}}$: the base value (i.e., the mean model output across all observations); $\varphi_{\text{j}}$: the contribution of feature j to the deviation from the base value.The larger the magnitude of $\left|\varphi_{\text{j}}\right|$, the greater the influence of that feature on the prediction.

## 3. Results

### 3.1. Model performance evaluation

To predict perceived walkability based on visual features, we developed separate Random Forest models for tourists and residents across five perceptual dimensions. Each model was trained using 80% of the images as the training set and 20% as the test set. Five-fold cross-validation was conducted to determine the optimal parameters. Table 1 presents the performance metrics for each model, including the number of optimal estimators (Best_n_estimators), coefficient of determination (R²), root mean square error (RMSE), mean absolute error (MAE), and out-of-bag (OOB) error. The results show that all models achieved R² values above 0.7, indicating good predictive power in estimating perceptual scores from SVIs. Notably, the tourist models demonstrated superior explanatory power in the dimensions of comfort (R² = 0.88) and pleasure (R² = 0.79), compared to the resident models (R² = 0.78 and 0.72, respectively). This suggests that tourists’ are more strongly aligned with visual features extracted from the images. In the convenience dimension, both the tourist (R² = 0.87) and resident (R² = 0.84) models performed well, reflecting the strong influence of functional street elements on perceived accessibility across user groups. In contrast, the resident models showed lower R² values in the sociability and safety dimensions, with slightly higher performance in the tourist models. This may be due to the greater subjectivity and complexity of residents’ social and safety perceptions, which are less easily captured by visual features alone.

**Table 1:** Random Forest model performance for residents and tourists.

Group	Indicator	Best_n_estimators	MAE	RMSE	OOB_Error	OOB_RMSE	R2_Score
Tourist	Safety	144	2.06	3.65	2.19	3.79	0.80
	Convenience	56	1.99	3.34	2.29	3.79	0.87
	Sociability	138	3.42	5.71	3.17	5.47	0.72
	Comfort	97	1.94	3.41	1.89	3.48	0.88
	Pleasure	199	2.74	4.88	2.94	4.96	0.79
Resident	Safety	181	2.37	4.10	2.19	3.67	0.73
	Convenience	57	2.38	3.63	3.04	4.86	0.84
	Sociability	56	1.58	3.11	1.87	3.69	0.71
	Comfort	141	2.49	3.93	2.60	4.42	0.78
	Pleasure	111	2.26	4.12	2.36	4.10	0.72

### 3.2. Descriptive statistics of perceptual scores

#### 3.2.1. Construction and validation of the composite perceptual score.

To unify the evaluation of street perception quality across different dimensions and support subsequent spatial analysis and regression modeling, we conducted separate factor analyses for the residents and tourists to derive the weights of each dimension contributing to the composite perceptual scores. Before extracting latent factors, we assessed sample adequacy and structural validity. The Kaiser-Meyer-Olkin (KMO) values were 0.864 for tourists and 0.897 for residents—both exceeding the 0.8 threshold—indicating strong sampling adequacy and good inter-variable cohesion. Bartlett’s test of sphericity reached statistical significance (p < 0.001), confirming sufficient correlation among variables to proceed with factor extraction. Table 2 presents the factor loadings and normalized weights for each perceptual dimension. The results show that the safety dimension consistently had the highest loading for both groups, indicating its dominant contribution to overall perceptual quality. Although the factor loadings were negative due to the orientation of the extracted components, only their absolute magnitudes were used for computing the normalized weights. Tourists assigned greater weight to safety, convenience, and pleasure, while residents emphasized safety and comfort. These findings reflect differing perceptual priorities shaped by users’ roles and spatial experience. The final composite perceptual scores was computed as the weighted sum of scores across the five perceptual dimensions, with weights derived from the respective factor loadings.

**Table 2:** Factor loadings and normalized weights for composite perceptual score among residents and tourists.

Indicator	Factor_Loading_Tourist	Weight_Tourist	Factor_Loading_Resident	Weight_Resident
Safety	−0.926	0.21	−1.228	0.294
Convenience	−0.902	0.204	−0.682	0.164
Sociability	−0.855	0.194	−0.551	0.132
Comfort	−0.864	0.195	−0.875	0.21
Pleasure	−0.914	0.207	−0.631	0.151
Overall KMO/ Bartlett	KMO = 0.864, Bartlett p = 0.000		KMO = 0.897, Bartlett p = 0.000	

#### 3.2.2. Comparative analysis of perceptual scores between tourists and residents.

**Fig 4:**
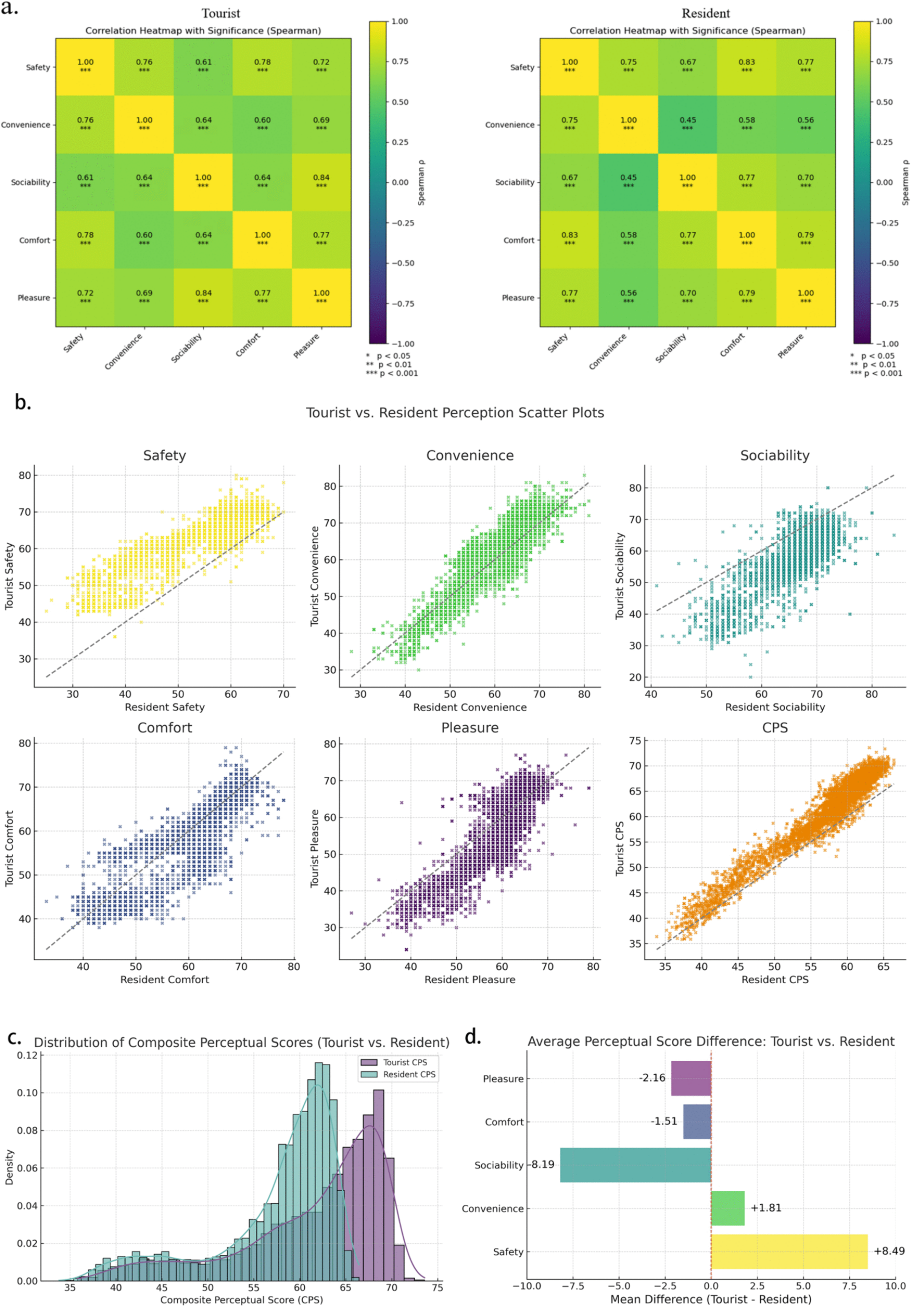
Statistical comparison and perceptual score distribution (tourists vs. residents).

Before comparing perceptual differences between residents and tourists, we first examined the internal correlations among the five perceptual dimensions to clarify their structural relationships. Spearman correlation matrices (Fig 4a) show consistently positive associations across all dimensions for both groups. For residents, correlation coefficients ranged from 0.45 to 0.83, with the strongest associations observed between safety–comfort (ρ = 0.83) and pleasure–comfort (0.79). For tourists, correlations ranged from 0.60 to 0.84, with particularly strong associations between pleasure–sociability (0.84) and safety–convenience (0.76). All correlations were statistically significant (p < 0.001). These results indicate that while the five perceptual dimensions share common experiential components—such as the tendency for safer streets to also be perceived as more comfortable or pleasant—they remain conceptually distinct and suitable for subsequent group comparisons and modeling. The slightly higher correlations among tourists suggest that tourists tend to form more holistic impressions of the street environment, whereas residents differentiate more clearly among functional and experiential attributes.

To further examine the differences in how tourists and residents perceive the urban walking environment, this section compares the two groups across the five perceptual dimensions as well as the composite perceptual scores. First, the scatter plot (Fig 4b) shows that tourists generally rated safety and convenience higher than residents, with most data points distributed above the y = x reference line. This indicates that tourists tended to evaluate the fundamental functional attributes of streets more positively. In contrast, residents gave notably higher scores in the dimensions of pleasure and sociability, suggesting a greater sensitivity to emotional connection and social atmosphere. The scores for comfort were relatively similar between the two groups, with residents scoring slightly higher. The scatter plot of the composite perceptual scores also reveals that tourists’ scores were more concentrated in the high-value range, while residents’ scores were more dispersed, with some street segments receiving significantly low ratings. Kernel density curves in Fig 4c further confirm this pattern: tourist composite perceptual scores scores exhibit a right-skewed distribution with a higher mean, reflecting an overall more positive and consistent attitude. Resident scores show a flatter, slightly left-skewed distribution, indicating greater diversity and criticality in their evaluations. Fig 4d compares the average scores across dimensions. Tourists rated safety and convenience higher than residents by 8.49 and 1.81 points, respectively, whereas residents scored higher in comfort (+1.51), pleasure (+2.16), and sociability (+8.19). These findings indicate that tourists tend to assign higher scores in safety and convenience, which may reflect a more general or less critical perception possibly due to limited familiarity with local conditions. Meanwhile, residents gave higher ratings for comfort, pleasure, and sociability, suggesting a more nuanced evaluation possibly shaped by daily usage experiences and social interactions in these spaces. However, these patterns do not necessarily imply that either group prioritizes specific dimensions in their judgment process.

**Fig 5:**
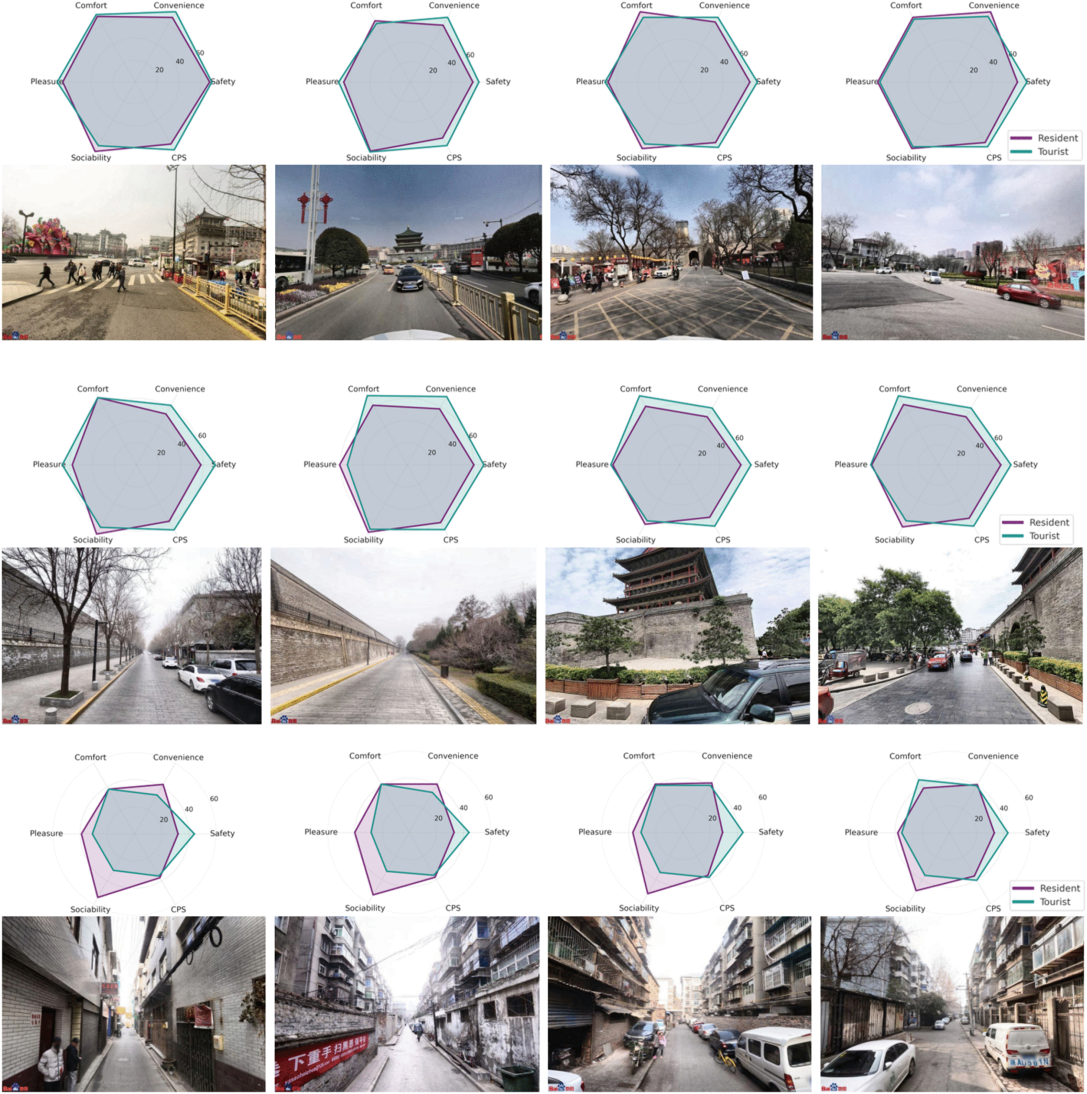
Comparison of five-dimensional perceptual scores between residents and tourists across typical street view samples.

To further illustrate the divergent perceptual structures of residents and tourists, we selected several representative street view samples and visualized their scores across the five perceptual dimensions and the composite perceptual scores using radar charts (Fig 5). The results show that tourists tended to assign higher pleasure and sociability scores to spaces with open views and strong cultural character, such as the areas in front of the city wall and Drum Tower squares. In contrast, residents gave higher scores in comfort and convenience to more livelihood-oriented streets characterized by rich everyday activities. Additionally, some street scenes received significantly higher ratings from tourists than from residents, highlighting discrepancies in cognitive structures within the same spatial context. These findings, at the individual sample level, support the earlier statistical results: tourists tend to favor spaces with open views, striking visuals, or culturally iconic elements, while residents place greater emphasis on functionality and a sense of belonging.

### 3.3. Spatial analysis of perceptual scores between tourists and residents

#### 3.3.1. Descriptive spatial statistics.

**Fig 6:**
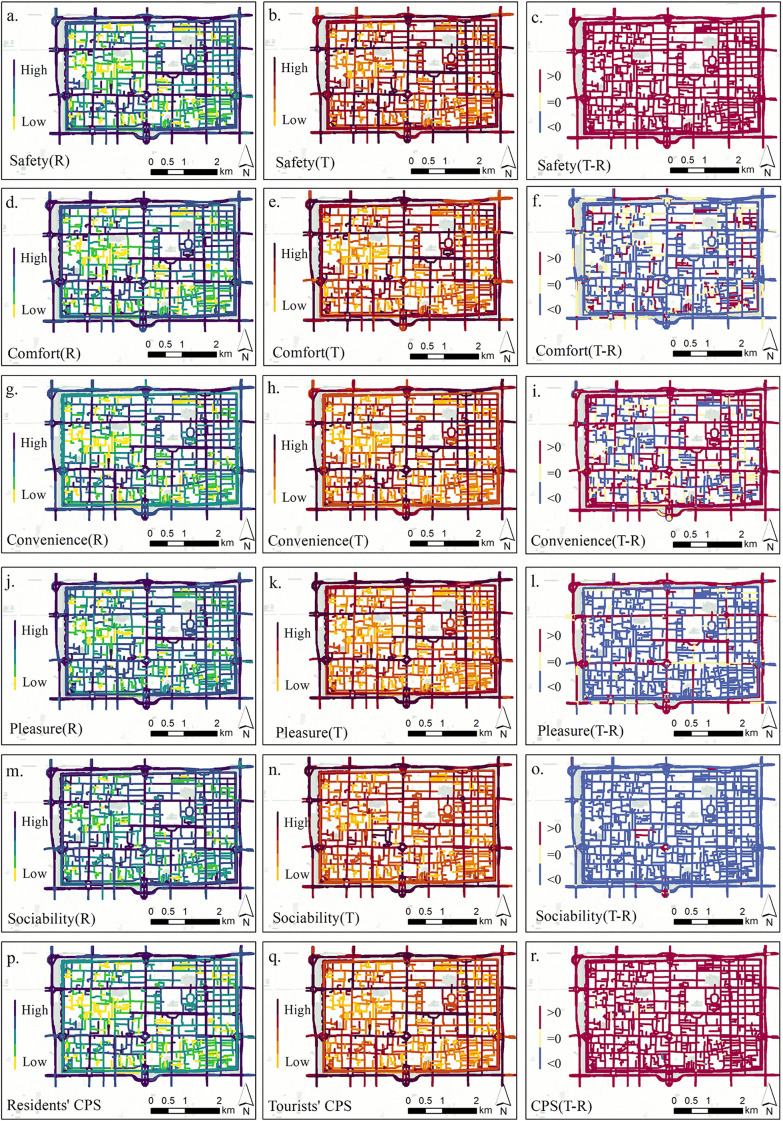
Spatial distribution and perceptual score differences between residents and tourists across five dimensions and composite perceptual scores.

To identify spatial variations in street perception between residents and tourists, this study mapped the scores of the five perceptual dimensions as well as the composite perceptual scores across the study area (Fig 6). Each column of the map displays the spatial distribution of residents’ scores (R), tourists’ scores (T), and the difference between the two (T–R), respectively, allowing a visual comparison of both perception levels and spatial preferences. Overall, the spatial patterns of perceptual scores show a certain degree of similarity between the two groups, with high values concentrated along urban main roads, cultural landmarks, transport hubs, and commercial pedestrian streets—forming a typical ‘central axis high-value zone.’ However, tourist scores exhibit a stronger spatial clustering, highlighting preferences for visual salience and destination landmarks. In contrast, residents’ scores are more widely distributed, indicating heightened sensitivity to everyday activity routes and familiar environments.

The difference maps reveal spatial nuances in perception divergence. For safety (Fig 6c) and composite perceptual scores (Fig 6r), tourists consistently scored higher than residents across nearly all areas, suggesting a stronger immediate sense of safety from visual stimuli. In contrast, residents, likely influenced by daily lived experiences, exhibited a more cautious assessment. For comfort (Fig 6f) and sociability (Fig 6o), spatial differences were bidirectional: residents gave higher ratings along commuting corridors, whereas tourists responded more positively in tourism hotspots such as Huimin Street and the Drum Tower area—indicating a preference for human presence and atmospheric vibrancy. In terms of convenience (Fig 6i) and pleasure (Fig 6l), tourists scored higher around subway stations, main roads, and cultural landmarks, demonstrating reliance on accessibility and visual attraction. Meanwhile, residents favored areas with dense local services.

#### 3.3.2. Spatial autocorrelation analysis.

To further examine whether perceptual scores from residents and tourists exhibit spatial clustering differences, we employed both global and local spatial autocorrelation methods (Global Moran’s I and Local Moran’s I) to evaluate the composite perceptual scores for the two groups. A Manhattan distance-based spatial weight matrix was constructed for the analysis. The results reveal that both groups exhibit significant positive spatial autocorrelation: the Global Moran’s I for residents was 0.484, and for tourists, it was 0.597, both statistically significant at p < 0.001. This indicates that high or low perceptual scores tend to cluster spatially. Notably, tourists showed stronger spatial clustering than residents, suggesting that their perceptual evaluations are more strongly influenced by structural spatial elements such as street axes and visual corridors. In contrast, residents’ perceptions are shaped by personal experience and local context, leading to more pronounced local heterogeneity.

**Fig 7:**
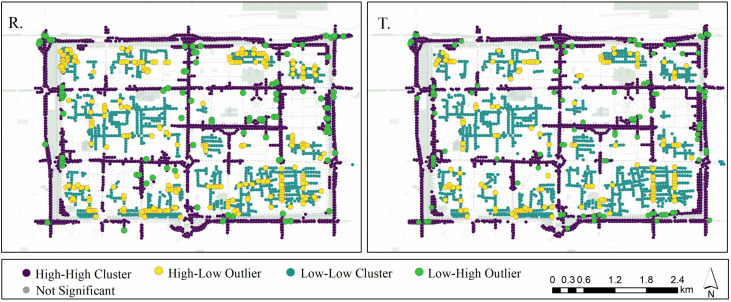
Local spatial autocorrelation of composite perceptual scores: Cluster and outlier types (Residents vs. Tourists).

In the local spatial autocorrelation analysis (Fig 7), we further identified street-level clusters categorized as High–High, Low–Low, and spatial outliers (High–Low and Low–High). The High–High clusters for residents were primarily located in areas such as Nanyuanmen, Shuyuanmen, and Xishaomen, where local life functions are dense. This highlights residents’ strong recognition and appreciation for their everyday living environments. For tourists, High–High clusters were concentrated around landmark nodes and tourism corridors such as the Bell Tower, Drum Tower, and Beidajie, indicating a preference for ‘recognizable spaces.’

Regarding spatial outliers, the number of High–Low and Low–High segments was significantly higher in the residents, indicating greater sensitivity to environmental transitions. Residents were more sensitive to detect disruptions in spatial continuity and discrepancies in place identity. These findings align with the prior spatial distribution analysis and reinforce the notion that tourist perceptions are primarily shaped by open views, striking visuals, or culturally iconic elements, while residents interpret streets based on lived experience, functional compatibility, and neighborhood familiarity. The interplay of symbolic cognition and functional cognition thus jointly shapes urban street perception.

### 3.4. Exploration of influencing factors

#### 3.4.1. Interpretable linear modeling: Elastic Net Regression and SHAP analysis.

To reveal the dominant role and directional influence of street-level semantic visual features in shaping composite perceptual scores, ENR models were separately constructed for residents and tourists, and interpreted using the SHAP algorithm to identify the marginal contributions and relative importance of each input variable. Both models were implemented using the scikit-learn library, with key hyperparameters set as follows: regularization strength α = 0.01, L1/L2 mixing ratio = 0.7, maximum number of iterations = 5000, and random seed = 42. Model training followed an 80/20 train-test split strategy. The modeling results demonstrate strong predictive performance for both models: the resident model achieved an R² of 0.832 with an RMSE of 2.269, while the tourist model achieved an R² of 0.860 with an RMSE of 3.001. These results indicate that visual features extracted from SVI can effectively explain over 80% of the variance in composite perceptual scores.

**Fig 8:**
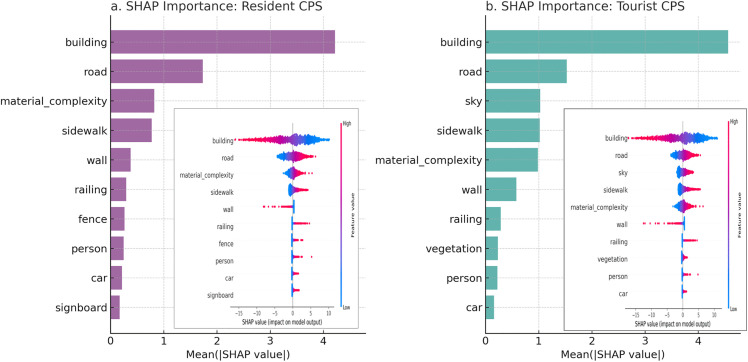
SHAP explanations of Elastic Net Regression models for composite perceptual scores (resident vs. tourist).

As shown in Fig 8, SHAP analysis revealed the directional effects of key visual features on composite perceptual scores. For both resident and tourists, building and wall emerged as significant negative predictors, indicating that highly enclosed or heavily built-up street environments may suppress positive perceptual experiences. This finding is consistent with previous studies [56,57]. Aside from these two negative features, the remaining variables generally showed positive associations with composite perceptual scores in both models. However, the ranking structures of feature importance differed substantially between the two groups. In the resident model, the top three positively contributing features were road, material_complexity, and sidewalk. This suggests that residents are more sensitive to the continuity of walking paths, the tactile and sensory quality of street materials, and the usability of pedestrian infrastructure. In contrast, in the tourist model, road was followed by sky as the second most influential feature, with sidewalk, material_complexity, and vegetation also showing strong positive contributions. These results suggest that tourists tend to favor visually open and green street environments. Notably, the presence of sky and vegetation as top-ranked features in the tourist model indicates a perceptual preference for spatial openness and environmental vividness, whereas the resident model places greater emphasis on practical walkability and material quality.

#### 3.4.2. Non-linear modeling with XGBoost and SHAP.

To identify the nonlinear relationship between street-level semantic visual features and composite perceptual scores, this study constructed XGBoost regression models and applied the SHAP algorithm to interpret model outputs, highlighting the directional effects and relative importance of key features. Both models were configured with identical hyperparameters (n_estimators = 200, max_depth = 3, learning_rate = 0.03). To mitigate overfitting, subsampling of training samples (subsample = 0.8) and feature columns (colsample_bytree = 0.8) was applied. Model performance was evaluated using an 80/20 train-test split and five-fold cross-validation, with MAE, RMSE, and R² as the evaluation metrics (Table 3). The results demonstrate that both models achieved high predictive accuracy, with R² values exceeding 0.98, indicating that semantic visual features effectively explain the perceptual differences between residents and tourists.

**Table 3:** Model performance comparison of XGBoost regression for resident and tourist composite perceptual scores predictions.

Metric	R_Train	R_Test	R_Difference	T_Train	T_Test	T_Difference
**MAE**	0.535	0.594	0.059	0.627	0.685	0.058
**RMSE**	0.697	0.793	0.096	0.837	0.924	0.087
**R** ^**2**^	0.989	0.986	−0.003	0.988	0.986	−0.002
**CV R**^**2**^ **(Mean)**			0.984			0.983

**Fig 9:**
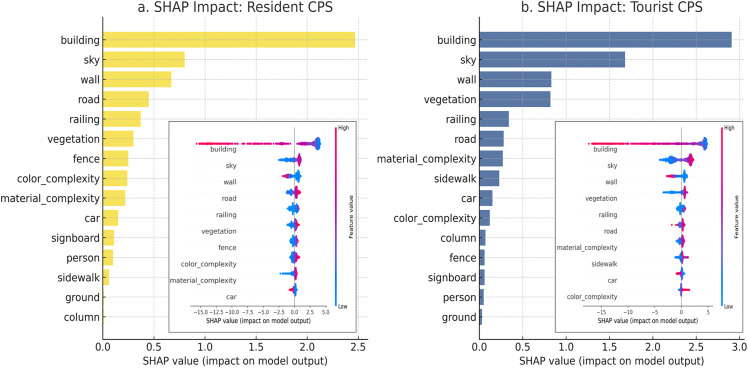
SHAP feature explanations of XGBoost regression models for residents and tourists’ composite perceptual scores.

As shown in Fig 9, in the resident model, the strongest negative features were building and wall, accompanied by a group of minor negative contributors collectively referred to as sum of other features. These features are mostly related to street enclosure, high proportions of solid mass, and a sense of spatial oppression, suggesting that residents are more likely to form positive perceptions in open and visually permeable street environments. Positive contributors included sky, road, railing, and vegetation; however, their SHAP values were more dispersed, indicating relatively mild influences.

The tourist model displayed a similar pattern of negative impact, primarily driven by building, wall, and car. However, its positive features such as sky, vegetation, road, and railing showed significantly stronger importance. Notably, the SHAP values for sky and vegetation were markedly higher than in the resident model, indicating that tourists are more sensitive to spatial openness and visible greenness. This preference highlights the tourists’ reliance on immediate visual stimuli and landscape – oriented features when perceiving urban streets. Additionally, in the resident model, the sum of other features exhibited an overall negative effect, implying that minor elements of the streetscape-possibly due to cluttered layouts or loose spatial organization – may reduce overall perceptual quality. In contrast, the tourist model demonstrated a more focused ranking of features, emphasizing visual salience, greenness, and openness. Meanwhile, the resident model reflected a more intricate cognitive mechanism, indicating a stronger dependence on long-term experiential knowledge related to spatial integrity and interface continuity.

**Fig 10:**
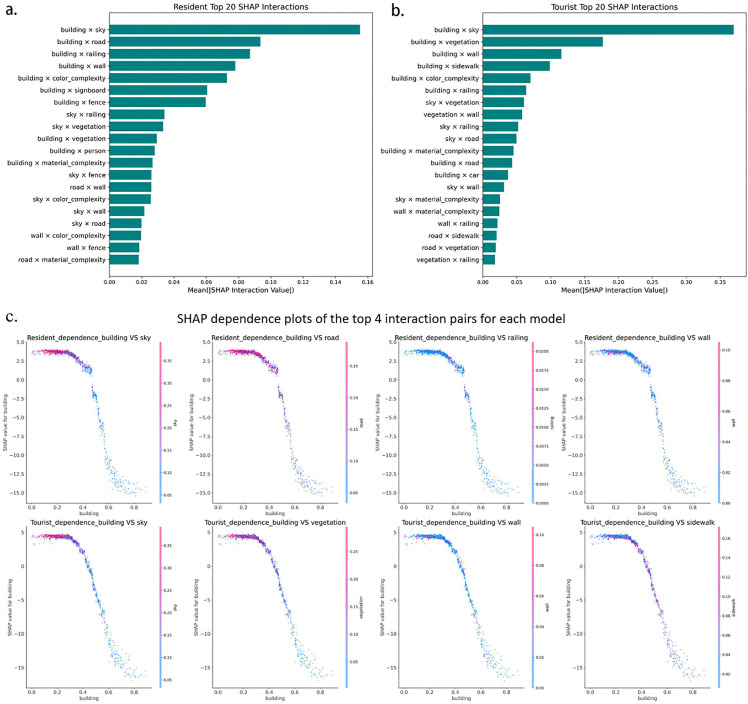
SHAP-based interaction analysis of resident and tourist perceptions.

To further explore the interaction effects among semantic features on perception scores, this study applied the shap_interaction_values function provided by SHAP to the trained XGBoost models. All pairwise interactions between semantic features were calculated, and the top 20 feature combinations were ranked based on the mean absolute interaction values (Fig 10a, b). For each group, the four most influential interaction pairs were visualized using dependence plots (Fig 10c). In the resident model, the combinations of building with sky, road, railing, and wall exhibited the strongest interaction effects. As the proportion of building increased beyond 0.4, its SHAP value dropped sharply, especially when the proportions of sky or road were low. This indicates that enclosed environments with high building density are perceived more negatively by residents. In the tourist model, the strongest interactions involved building paired with sky, vegetation, wall, and sidewalk. Tourists also responded negatively to high building density, particularly when both sky and vegetation proportions were low. Conversely, the negative impact of building was mitigated in contexts where sky and vegetation were more abundant.

## 4. Discussion

### 4.1. Interpreting perceptual differences in urban walking environments

Based on the fitting results of machine learning models and spatial distribution analysis, this study empirically reveals significant perceptual differences between tourists and residents in urban street environments, particularly in the dimensions of safety, comfort, and sociability. Overall, tourists tend to give higher scores, especially favoring spaces with rich cultural atmosphere and high visual openness. In contrast, residents show more differentiated evaluations across dimensions, exhibiting higher sensitivity. These differences can be attributed to the distinct roles each group plays in urban space. Tourists, as short-term experiencers, are more likely to have their perceptions triggered by visual cues, aesthetic appeal, and cultural symbolism in the environment [58–60], forming an overall satisfaction through rapid judgment. Residents, on the other hand, establish continuous and routine relationships with the streets, and their perceptions emphasize functionality, safety, and long-term usability [42,61]. As such, tourists’ high ratings are concentrated in main corridors and landmark nodes, while residents demonstrate stronger perceptual responses along everyday routes and backstreets. These findings support the development of a differentiated cognitive framework for street perception that explicitly accounts for user identity. Rather than treating perception as a universal response to visual stimuli, this study shows that perceptual evaluations are structured by the spatial roles and lived experiences of distinct user groups. By foregrounding the contrast between visual symbolism (tourists) and functional familiarity (residents), this framework contributes to urban perception theory by integrating perceptual diversity and socio-spatial roles into cognitive modeling.

### 4.2. Driving forces of spatial and semantic differences

According to the spatial statistical results, the distribution of composite perceptual scores between residents and tourists exhibits clear patterns of clustering and displacement. Tourists’ high-score zones are concentrated around cultural landmarks, plaza spaces, and main visual corridors, reflecting a strong preference for recognizability-driven spatial features. In contrast, residents’ high-score areas are more dispersed, covering daily-life alleys and service-dense neighborhoods, indicating a use-experience-oriented cognitive structure. This spatial mismatch suggests that different user groups hold divergent definitions of what constitutes a good street.

The SHAP analysis provides a semantic-level explanation of this divergence. In the Elastic Net model, road, sidewalk, and material_complexity exhibit high linear effects in resident perception, emphasizing the importance of connectivity, accessibility, and material clarity in everyday use. While these elements still exert a positive influence in the tourist model, their contributions are diminished and are significantly replaced by sky (visual openness) and vegetation. These natural and visual features demonstrate nonlinear enhancement effects in the XGBoost model, indicating an ‘aesthetic threshold’ for tourists [34] – only when these features reach a certain proportion do they significantly boost overall perception. This suggests that tourists rely more on visual salience, while residents base their judgments on lived experience and multidimensional assessments [1,62]. SHAP interaction analysis further revealed that building frequently participated in the most influential feature pairs, yet its perceptual impact depended on the surrounding context. Negative effects were stronger in visually enclosed or low-greenery environments, particularly for tourists. These findings highlight the importance of co-occurring semantic features and suggest that perception is shaped not only by individual elements but also by their spatial and visual combinations.

### 4.3. Implications for inclusive street design

The findings of this study highlight that urban street design should not rely on the assumption of an average user, but must address the diverse roles, psychological cognition, and spatial interactions of different user groups. This aligns with recent consensus on social equity in public space research. For instance, Perera et al. have noted a significant discrepancy between planners and communities in public space design ideologies, calling for strategies that balance equity with local narratives to bridge cognitive gaps [63]. Suarez et al. systematically reviewed the practices of diversity, equity, and inclusion in green space planning, stressing the importance of incorporating marginalized groups into spatial policymaking [64]. Remesar, using Barcelona as a case study, advocated co-design processes involving diverse stakeholders as effective means to realize spatial justice [65].

Against this theoretical backdrop, this study proposes a data-driven empirical basis for perceptual equity-oriented street design. Specifically, resident-dominated streets should prioritize continuity of pedestrian flow, safety, and interface friendliness to enhance spatial stability and comfort. Residential neighborhood streets should focus on social interaction, shading conditions, and walkability. Tourist-dense streets should emphasize visual accessibility and cultural symbolism to enhance legibility and iconicity. Mixed-use streets should incorporate symbolic nodes [66] to respond to the emotional expectations of both residents and tourists.

By leveraging the revealed variable rankings and perceptual preference heterogeneity, urban designers can develop fine-grained intervention strategies based on ‘user personas,’ advancing the paradigm shift from universal applicability to context-sensitive adaptation in street design.

### 4.4. Limitations and future directions

Despite its strengths in high-precision modeling and explainability, this study has several limitations. First, this study focuses on the Mingcheng District of Xi’an, a historic urban core rich in cultural heritage. While this offers a meaningful context for exploring street perception, it may limit the generalizability of findings to other cities. Additionally, although the analysis emphasizes environmental and visual features from SVIs, broader contextual factors—such as urban policy, tourism strategies, or cultural narratives—were not explicitly considered, which may particularly affect perception in heritage-rich areas. Second, the analysis is based on static SVIs that capture a single temporal moment, without accounting for diurnal or seasonal changes. However, perceived dimensions such as safety or comfort can vary significantly under different lighting or weather conditions, which may also contribute to perception gaps between residents and tourists. Third, the group classification distinguishes only between residents and tourists, without further segmentation by age, gender, or travel purpose, which may oversimplify heterogeneous user needs. Fourth, due to the reliance on publicly available SVIs, certain underground or visually enclosed spaces (e.g., tunnels, courtyards) may be underrepresented. Although such images were intentionally excluded to ensure visual clarity, this may introduce a subtle bias toward more accessible and tourist-friendly areas.

Future studies could address these limitations by expanding to multi-city comparisons, incorporating multimodal perception data (e.g., sound, smell, narrative text), and integrating eye-tracking or EEG technologies to better capture the design–cognition–behavior relationship. Large language models (LLMs) also offer potential to augment or validate perception data, especially where participant recruitment is constrained. Moreover, generative AI could simulate design interventions to explore causal effects more dynamically. As inclusive and intelligent governance gains momentum, understanding diverse urban experiences will be key. This study offers an initial framework integrating AI and perceptual diversity, paving the way for scalable and adaptable urban street assessments.

## 5. Conclusion

This study, grounded in SVIs, machine learning, and SHAP-based explainability, systematically reveals the perceptual heterogeneity between residents and tourists in urban street environments. Our findings demonstrate significant differences in cognitive preferences, spatial trajectories, and influencing variables across groups—residents rely more on structural and functional features, while tourists are notably driven by visual stimuli and symbolic spatial elements. The combined use of ElasticNet and XGBoost highlights a dual-track perceptual mechanism, encompassing structural stability and nonlinear threshold effects. Theoretically, the study expands the role–cognition model by emphasizing user differentiation in urban perception. Methodologically, it proposes a scalable and interpretable framework for perceptual evaluation that integrates street view imagery, semantic segmentation, spatial analysis, and explainable machine learning. Compared to traditional survey-based or black-box modeling approaches, this framework enhances cross-group comparability, spatial explicability, and model transparency. The framework is transferable to other urban contexts where comparable street view data and perception ratings are available, particularly for studies examining perceptual differences across user roles. Practically, the findings provide quantitative evidence and an operational basis for differentiated and inclusive street design, contributing to the advancement of equitable and livable urban environments.
